# Aryl-hydrocarbon receptor binding and the incidence of type 2 diabetes: the Brazilian Longitudinal Study of Adult Health (ELSA-Brasil)

**DOI:** 10.1186/s12940-020-00658-y

**Published:** 2020-10-12

**Authors:** Bruce B. Duncan, Cristina D. Castilhos, Paula A. Bracco, Maria I. Schmidt, Sora Kang, Suyeol Im, Hong-Kyu Lee, Álvaro Vigo, Youngmi K. Pak

**Affiliations:** 1 Postgraduate Program in Epidemiology and Hospital de Clínicas, Universidade Federal do Rio Grande do Sul, Ramiro Barcelos, 2600/514, Porto Alegre, RS 90035-003 Brazil; 2 Department of Neuroscience, Medical Research Center for Bioreaction to Reactive Oxygen Species and Biomedical Science Institute, School of Medicine, Graduate School, Kyung Hee University, Seoul, South Korea; 3 Department of Internal Medicine, College of Medicine, Eulji University, Seoul, South Korea; 4 Department of Physiology, College of Medicine, Kyung Hee University, 26 Kyungheedae-ro, Dongdaemun-gu, Seoul, 02447 South Korea

## Abstract

**Background:**

Persistent organic pollutants (POPs) may cause diabetes, in part through aryl hydrocarbon receptor (AhR) binding. Ensuing mitochondrial dysfunction is postulated to mediate this effect. We aim to investigate the association of POPs with incident diabetes indirectly by bio-assaying AhR ligand bioactivity and intracellular ATP level induced by participant serum samples.

**Methods:**

In incident case-cohort analyses of one ELSA-Brasil center, 1605 eligible subjects without diabetes at baseline had incident diabetes ascertained by self-report, medication use, OGTT or HbA1c at follow-up 4 years later. We assayed AhR ligand bioactivity (AhRL) and intracellular ATP content, the latter reflecting the presence of mitochondria-inhibiting substances (MIS), following incubation of recombinant mouse Hepa1c1c7 cells with participant sera for 71 incident diabetes cases and 472 randomly selected controls.

**Results:**

In multiply-adjusted proportional hazards regression analyses, those with above-median AhRL and below-median MIS-ATP had 69 and 226% greater risk of developing diabetes (HR = 1.69; 95%CI 1.01–2.83 and 3.26; 1.84–5.78), respectively. A strong interaction was seen between the two exposures (HR_high AhRL/low MIS-ATP vs. low AhRL/high MIS-ATP_ = 8.15; 2.86–23.2).

**Conclusion:**

The markedly increased incidence of diabetes seen in those with both higher AhR ligand bioactivity and increased mitochondrial inhibition supports the hypothesis that widespread POPs exposure contributes to the diabetes epidemic.

## Background

A slow-moving pandemic of diabetes has swept across the world over recent decades. Many studies have suggested the possibility that persistent organic pollutants (POPs), whose concentration in living beings has increased in many countries over this period, can be a causal factor of this pandemic [1–4]. Investigation of this hypothesis has been complicated by the multitude of POPs polluting the environment and difficulties in their collection and measurement, given that they are present in extremely small quantities.

We measured serum-induced aryl hydrocarbon receptor ligand-mediated luciferase bioactivity (AhRL) with transfected mouse Hepa1c1c7 cells [5]. The AhR is a cytosolic nuclear receptor that binds many xenobiotic ligands, including dioxins [6]. A variety of chemicals, including POPs and other endocrine disrupting or metabolism disrupting chemicals, are ligands of AhR. As such, AhRL is a surrogate biomarker of the level of exposure to the chemical mixture of aromatic hydrocarbon compounds, including POPs, in humans. Activation of the AhR may partially mediate POP effects [7]. The assay is inexpensive and in principal integrates in impact of multiple POPs and other metabolism disrupting chemicals in a single value.

Mitochondrial dysfunction has been proposed to cause diabetes [8] through induction of insulin resistance in both muscle and liver via a buildup of excess lipids as well as through a decrease in insulin secretion resulting from lower beta cell ATP content [9]. In parallel with the AhRL assay, we also applied a similar cell-based assay to indirectly detect the presence in participant serum samples of mitochondrial inhibiting substances (MIS) by measuring intracellular ATP content in serum-incubated cultured cells (MIS-ATP) [10]. Both AhRL and MIS-ATP, so measured, have recently been shown to be highly predictive of future diabetes [11].

The objective of this report is to investigate the ability of participant serum-induced AhR bioactivity and mitochondrial dysfunction to predict the development of diabetes in participants of the Rio Grande do Sul center of ELSA-Brasil, a cohort study of Brazilian civil servants initiated in 2008.

## Methods

The Brazilian Longitudinal Study of Adult Health (in Portuguese, Estudo Longitudinal de Saúde do Adulto or ELSA-Brasil) is a multi-center cohort study designed principally to investigate diabetes and cardiovascular disease. The details of the study, including design, eligibility criteria, sources and methods of recruitment, and measurements obtained have been described in detail elsewhere [12, 13]. At ELSA’s Rio Grande do Sul center, we enrolled university and university hospital employees (*n* = 2061) aged 35–74 years, between 2008 and 2010. Participants returned for follow-up examination from 2012 to 2014. At both visits, we obtained information by interview and measured weight, waist, hip, height and blood pressure following standardized protocols. All methods were performed in accordance with the relevant guidelines and regulations.

We performed a standardized 75 g oral glucose tolerance test (OGTT) in all participants without known diabetes utilizing an anhydrous glucose solution at baseline and follow-up visits [14] Plasma glucose was measured by the hexokinase method (ADVIA Chemistry; Siemens, Deerfield, Illinois). HbA1c, also obtained, was measured by high-pressure liquid chromatography (Bio-Rad Laboratories, Hercules, California), using a method certified by the National Glycohemoglobin Standardization Program. Blind replicates were measured for all analytes. The interclass correlation coefficient for glucose was 0.99 (95%CI 0.95–1.00) and 0.94 (95%CI 0.86–0.97) for glycated hemoglobin [15].

Diabetes was ascertained at baseline and follow-up based on self-report of a physician diagnosis of diabetes, use of medication for diabetes in the past 2 weeks, or laboratory values reaching the threshold for fasting plasma glucose (FPG; ≥7.0 mmol/L), 2-h plasma glucose during the OGTT (2 h PG ≥11.1 mmol/L), or HbA1C (≥6.5%; ≥47.5 mmol/mol) [14, 16, 17].

Of 2061 initial Rio Grande do Sul center subjects, 1605 were free of diabetes at baseline, returned for a follow-up visit, and had complete information to classify diabetes at baseline and follow-up. After exclusion of those with missing values at baseline for the covariates of interest (*n* = 23), we performed analyses on incident (*n* = 71) diabetes cases with available sample and on an accompanying 472 randomly selected controls from the cohort in a case-cohort design.

For these, we determined AhRL and MIS-ATP with the above-mentioned cell-based bioassays [10], both performed in Korea on blinded samples which had been collected at baseline and stored at -80 °C. In brief, the pGL4-DRE-luc(puro+)/pRL-mTK-transfected mouse Hepa1c1c7 cells were incubated with 10 μl (10% of culture media) serum samples for 24 h. DRE-dependent luciferase activity is produced by these cells as a function of AhR binding by substances in the sample. Before incubation, participant serum samples and charcoal-stripped human serum were prepared as described previously [10]. Serum-induced AhR ligand-mediated luciferase activity, normalized to Renilla luciferase activity, referred to as AhRL, was quantified as fold induction (FI), compared to the activity induced in the 10% charcoal-stripped human serum (CSS)-treated cells, set as 1. When the fold induction of AhRL is converted to its TCDD (2,3,7,8-tetrachlorodibenzo-p-dioxin) equivalent using the equation of the standard curve AhRL (FI) = 0.5279 x TCDD (pM) + 1.261 (*r*^*2*^ = 0.9692, *p* <  0.0001) [11], the median value of 2.04 FI is equivalent to 1.48pM of TCDD.

Similarly, to determine levels of mitochondria-inhibiting substances in serum samples, pRL-mTK-transfected Hepa1c1c7 cells were incubated with 10 μl serum from the samples for 48 h, and ATP-dependent luciferin-luciferase activities were determined to measure ATP content.

The interclass correlation coefficients for these determinations, based on blind replicate measurements of 50 participants, were 0.25 (0.17–0.38) for AhRL and 0.29 (0.08–0.52) for MIS-ATP.

To characterize the association between enhanced AhRL and MIS-ATP with incident diabetes, we first categorized the two exposures at their median values. Using the categories so derived, we then investigated risk for incident diabetes with proportional hazards regression. To permit adjustment for potential confounding factors despite our relatively small sample size, we utilized propensity scores obtained with logistic regression considering age, sex, family history of diabetes, hypertension, educational attainment, self-reported ethnicity, smoking status (current, former, and never), BMI and WHR. Next, we investigated the joint effect of AhRL and ATP inhibition on diabetes, when categorized, by combining the high/low categories and using as reference those with low AhRL and high MIS-ATP. Additionally, we investigated the continuous effects of the two exposures separately on diabetes using a restricted cubic spline approach performed with the RCS_REG macro [18]. We investigated, as a sensitivity analysis, adjusting the results alternatively for the number of cigarettes smoked per day instead of for smoking status. Due to missing values for that variable (32 control and 5 cases), we decided to use adjustment for smoking status in our main analysis.

All analyses were weighted with weight equal to one for diabetes cases and equal to the inverse of the sampling fraction for random sample controls, i.e., if the selected controls represented 5% of those without diabetes in the eligible sample, then each control had a weight equal to 20 [19]. All analyses were performed with the SAS 9.4 using proc survey to incorporate the respective weights of cases and controls.

## Results

Table 1 shows the distribution of select socio-demographic and clinical factors in a random sample and in those who developed diabetes during follow-up of the Rio Grande do Sul ELSA cohort. Incident cases had 6.2% lower MIS-ATP (*p* <  0.0001) and similar (5% greater) mean AhRL (*p* = 0.19). Additionally, they presented higher mean BMI and waist/hip ratio, were slightly older, and had lesser educational attainment and a more frequent family history of diabetes.

**Table 1:** Baseline descriptive data for the cohort random sample and for cases of incident diabetes. ELSA-Brasil Rio Grande do Sul Center, 2008–2010

Variable	Cohort Random Sample *N* = 472	Incident Cases *N* = 71	*p*-value*
	N(%) / Mean (SD)	N(%) / Mean (SD)	
AhRL (FI)	2.2 (0.6)	2.3 (0.6)	0.1933
MIS-ATP (%)	87.4 (9.8)	82.0 (9.9)	< 0.0001
Sex			
Female	268 (56.8%)	40 (56.3%)	0.9442
Schooling			
Incomplete High School	53 (11.2%)	11 (15.6%)	0.1989
Incomplete University Education	147 (31.1%)	27 (38.3%)	
Complete University Education	272 (57.6%)	33 (46.5%)	
Smoking			
Never	289 (61.2%)	43 (60.6%)	0.9294
Former	124 (26.3%)	18 (25.3%)	
Current	59 (12.5%)	10 (14.1%)	
Number of cigarettes (all participants)	0.9 (4.3)	2.4 (0.1)	0.0270
Number of cigarettes (among those who smoked)	13.5 (11.4)	32.0 (18.2)	0.0047
Family History of Diabetes			
Yes	154 (32.64%)	29 (40.9%)	0.1720
Race/Ethnicity			
Not white	103 (21.8%)	19 (26.8%)	0.3526
Age	53.1 (9.1)	53.9 (9.3)	0.4821
BMI (kg/m^2^)	26.5 (4.3)	28.5 (4.4)	0.0003
Waist/Hip Ratio	0.93 (0.08)	0.89 (0.09)	0.0002

*p-value are for comparisons between incident cases and the cohort random sample, calculated through t-tests for continuous variables and chi-square tests for categorical variables, using appropriate weighting

AhRL = aryl hydrocarbon receptor ligand-mediated luciferase bioactivity

FI = fold induction compared with that of control cells incubated with charcoal-stripped human serum

MIS-ATP = intracellular ATP content in serum incubated samples

% = % of ATP content present in control cells incubated with charcoal stripped serum

In crude analysis, those with above median AhRL (> 2.04 fold increase) demonstrated greater risk of developing diabetes (HR = 1.85; 95%CI 1.11–3.06), this risk being minimally affected by adjustment for confounding factors (HR = 1.69; 95%CI 1.01–2.83) (Table 2). Those with below median MIS-ATP (< 87.64% of charcoal stripped serum-treated control) had increased risk of developing diabetes, both in crude and adjusted analyses (HR = 3.62; 95%CI 2.06–6.38 and HR = 3.26; 95%CI 1.84–5.78, respectively). When adjusting for the number of cigarettes smoked instead of smoking status our results barely changed for AhRL (HR = 1.65; 95%CI 0.97–2.80 and deceased slightly for MIS-ATP (HR = 2.81; 95%CI 1.56–5.06).

**Table 2:** Risk^a^ of incident diabetes for those with above median values of aryl hydrocarbon receptor ligand-mediated luciferase bioactivity (AhRL) and intracellular ATP content (MIS-ATP) in serum incubated samples

	Crude	Adjusted
	HR (95%CI)	HR (95%CI)
AhRL (FI)		
< 2.04	1	1
> 2.04	1.85 (1.11; 3.06)	1.69 (1.01; 2.83)
MIS-ATP (%)		
> 87.64	1	1
< 87.64	3.62 (2.06; 6.38)	3.26 (1.84; 5.78)

^a^As determined through proportional hazards modeling for incident analyses, adjusted utilizing a propensity score based on age, sex, family history of diabetes, hypertension, educational attainment, self-reported ethnicity, smoking status, BMI and waist/hip ratio. FI = fold induction; % = % of ATP content present in control cells incubated with charcoal stripped serum

**Fig. 1:**
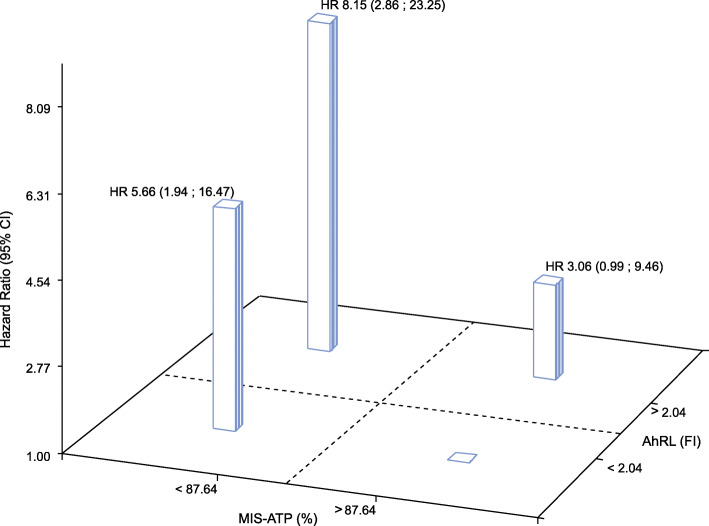
Adjusted association of the incidence of diabetes in the isolated and joint presence of above median aryl hydrocarbon receptor ligand-mediated luciferase bioactivity (AhRL) and below median intracellular ATP content (MIS-ATP) in serum-incubated cultured cells. FI = fold induction; % = % of ATP content present in control cells incubated with charcoal stripped serum (CSS)

Risk of diabetes for those with both high AhRL and low MIS-ATP, compared to those with both low AhRL and high MIS-ATP (Fig. 1) was very high (HR = 8.15; 95%CI 2.86–23.25), being slightly decreased when adjusting rather for the number of cigarettes smoked (HR = 6.9; 95%CI 2.4; 20).

**Fig. 2:**
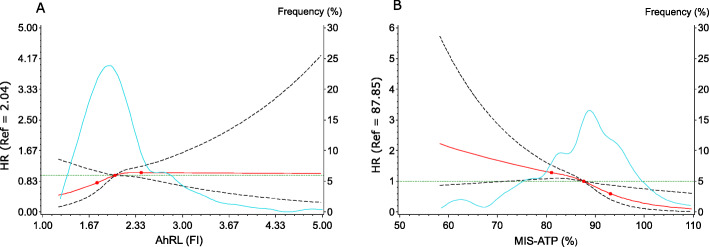
Adjusted associations of incident diabetes with aryl hydrocarbon receptor ligand-mediated luciferase bioactivity (AhRL, Panel **a**) and intracellular ATP content (MIS-ATP, Panel **b**) in serum-incubated cultured cells. Point estimates of hazard ratios (HR) at different levels of exposure (red lines) are shown with 95% confidence intervals (black dashed lines). The distributions of AhRL and MIS-ATP in the cohort random sample are superimposed (faint blue lines). The green lines indicate a HR = 1.0. FI = fold induction; % = % of ATP content present in control cells incubated with charcoal stripped serum (CSS)

Analysis via restricted cubic spline regression of the AhRL associations, when this serum-induced bioactivity was expressed continually (Fig. 2), showed a rise in risk of diabetes along the first half of the relevant range of AhRL in the random sample, with a plateauing at greater values, without statistical significance. The association of increased risk with decreased MIS-ATP was relatively linear along the whole random sample distribution, with the zone of 95% confidence showing statistical significance throughout almost all of the relevant range.

## Discussion

Our findings of associations of increased AhRL and decreased MIS-ATP with the development of diabetes, with a large positive interaction between the two exposures, are in consonance with the hypothesis that exposure to persistent organic pollutants is a contributing cause to the current diabetes epidemic. The associations, with the exception of the joint effect of both high AhRL and low MIS-ATP (HR = 7.23), are small. This fact, coupled with the small size of our sample, limits the strength of our overall findings. Nonetheless, being prospective in nature and with findings similar to those recently reported in a Korean cohort [11], our study presents important evidence to support a causal role of these pollutants in the current diabetes pandemic.

A marked ecologic correlation exists between the increase in POPs in the environment and the increase in diabetes in the population [1]. The diabetes pandemic affects both rich and poor, urban and rural, and has spread across the globe. Thus a broad exposure such as that of POPs, which is worldwide and has grown over the past 70 years, could well help explain the presence and extent of the pandemic. Moreover, the recent decrease in the overall incidence of diabetes in many high-income countries [20] is consistent with the POPs hypothesis, as many of the major monitored POPs have decreased in several localities of the world’s northern hemisphere [21, 22], where most of the countries with declines are located.

That POPs can cause diabetes is biologically plausible. Many studies in animals and involving small human samples show deleterious effects of POPs: greater levels have been associated with obesity, impaired fasting glucose, impaired glucose tolerance, and insulin resistance [1, 3, 23].

Many epidemiologic studies in different populations and using different study designs have shown associations between POPs and diabetes. Most of these have some limitation in their ability to contribute to the question of causation in general populations – being cross-sectional in nature, investigating geographically delimited populations having much higher levels of exposure, or producing weak associations of limited statistical significance. Additionally, given the myriad of different POPs, the interpretation of positive associations of some POPs in the context of multiple hypothesis testing is difficult. In fact, in one study [24], only a non-statistically significant 80% increased risk was found with dioxin-like POPs, while an almost 5 fold increased risk was found with other POPs. All these details may be making it hard to see the forest for the trees [3, 4].

Several reviews have summarized epidemiologic studies in general populations. More recent and robust cross-sectional studies have shown a much-increased prevalence of diabetes (prevalence ratios> 10) in the presence of obesity combined with elevated summary measures integrating levels of multiple POPs. Among the more convincing findings arising from cohort studies, the Coronary Artery Risk Development in Young Adults and the Prospective Investigation of the Vasculature in Uppsala Seniors, using summary measures of exposure to multiple POPs, showed that those with ranges of exposure to mixtures of POPs had 3–5 times the risk of developing diabetes when compared to those with low levels. However, not all studies have produced positive findings, and relatively few prospective epidemiologic investigations have been performed [3, 23, 25].

As recently suggested, to establish a firm link between POPs and diabetes, the scientific community must respond to three questions [26]: 1. Can we predict the net effect of endocrine disrupting chemical mixtures in the real world? 2. Can non-monotonic dose-response relationships be validly evaluated in human studies? and 3. Can exposure to endocrine disrupting chemicals with short half-lives be reliably assessed in humans? Investigation of AhRL and MIS-ATP in human serum permits a positive response to these questions.

With respect to the first and third questions, AhRL is logically a good surrogate for the net effect resulting from the presence of multiple POPs, independent of their half-life, and mitochondrial dysfunction may be a major mediator of their net effect.

The second question relates to the fact that an inverted, U-shaped dose-response relationship has frequently been found in the association of increasing levels of POPs with diabetes. However, a dose-response curve which increases at low levels and then peaks and stabilizes or even decreases at higher levels is well described in general with respect to biologic functions [23] and specifically for the AhR [27]. Greater AhRL, for example, generates the AhR repressor (AhRR) protein, a ligand-independent AhR-like protein that constitutively translocates into the nucleus where it acts to inhibit nuclear responses to AhR binding [28]. Our spline analyses permit visualization of the dose-response relationship we found – an increasing risk only across the first half of the relevant range of AhRL, consistent with this understanding of AhR action. This pattern of association strengthens our findings.

Considerable literature supports mitochondrial dysfunction as a causal pathway to diabetes. Though the immediate causes of type 2 diabetes – insulin resistance and a progressive decrease in insulin secretion by the beta cell – have long been identified, the pathophysiologic processes which translate environmental and behavioral exposures into declines in insulin sensitivity and secretion are not clear. Within this context, recognition of the possible role of the mitochondria has grown over recent years. Mitochondrial dysfunction has been linked to a 60% decrease in muscle insulin sensitivity in healthy, young, lean insulin-resistant offspring of parents with diabetes [29]. A cogent argument has been made that when the mitochondria function poorly, lipid products accumulate in cells, leading to lipid toxicity, which produces insulin resistance in cells metabolizing glucose. Perhaps more importantly, worsened mitochondrial function in beta cells results in decreased ATP content which in turn leads to decreased insulin secretion [8, 9].

Greater binding to the AhR, called at times the dioxin receptor, has been shown to be both an excellent surrogate for POP levels in sera ^5,21^ and a good surrogate for POP exposure. Such binding has also been associated with higher levels of insulin resistance and glycemia [5, 8, 30]. Yet the pathophysiologic steps through which such binding can lead to diabetes are less clear. The principal role defined to date for the AhR, a ligand-activated transcription factor, is to respond to the presence of xenobiotics. However, a broader understanding of its function includes regulation of a wide variety of endogenous physiological functions and processes, adaptive responses, and toxic effect [27]. The AhR plays a major role in altering immunologic and metabolic actions in response to the presence of xenobiotics, that is to say, adjusting cellular function in the presence of an altered environmental milieu. One documented adjustment is through altered mitochondrial function [8]. Although much remains to be learned about the relationships between POPs, AhRL and mitochondrial function, AhRs are found within the mitochondria [7], and several studies in tissues and in animal models have shown altered mitochondrial function in the presence of POPs [8].

Following this reasoning, the presence of POPs such as dioxins, not present during the evolution of these cellular mechanisms, may produce a pathological disruption of metabolism involving both insulin resistance and beta cell decline, apparently partially mediated by the AhR and at least partially the result of mitochondrial dysfunction. Our data, together with others [5, 11, 30], who have used the same cell-based assays here employed provide epidemiologic evidence produced in free-living populations which validates previous basic science and clinical research on the role of mitochondrial dysfunction in the pathophysiology of diabetes [5, 7, 25, 26, 29, 30], especially in the presence of greater AhR activation, thus strengthening the contention that POPs cause diabetes and providing a causal pathway for their action.

Our study has important limitations. First, with only 71 incident cases of diabetes, our estimates of association are statistically fragile. Second, our small sample size imposes limitations on our ability to investigate the potential role of specific confounders. However, in this regard, our propensity score analyses suggest that little confounding is present, consistent with the presence of a broad environmental exposure. Third, AhRL may reflect the presence of agonists other the POPs in the serum of our participants, and the lower ATP levels which associated with incident diabetes may have occurred via mechanisms unrelated to POPs or AhRL. Finally, the cell-based bioassays we employed to estimate POP exposure and measure mitochondrial function have poor reproducibility, as documented by the very low interclass correlation coefficients in reliability analyses. In this regard, more reproducible measures could potentially produce much greater associations. Hopefully our findings, together with the recent ones of others [11] will lead to advances in assay techniques which will produce more precise, reproducible, and readily available measurements of the phenomena we analyzed.

Our study also has several strengths. Laboratory analyses of AhRL and MIS-ATP, though performed in the same laboratory as those of the Ansung cohort, were conducted blinded to any knowledge of participant characteristics or glycemic status. Additionally, ELSA-Brasil is a major independent cohort study undertaken in a very different society halfway around the globe from the Korean Ansung cohort. Together with the findings of the Ansung cohort, in which those with greater than median AhRL had nearly 8 times the risk, those with less than median MIS-ATP approximately 4 times the risk and those with both high AhRL and low MIS-ATP had approximately 21 times the risk of incident diabetes of those in the low risk categories of these measures [11], they strengthen the hypothesis that exposure to POPs causes diabetes.

## Conclusion

We have demonstrated in a small case-cohort sample of middle-aged and elderly Brazilians that greater AhRL, a surrogate for the level of POPs present in the baseline serum of participants, in the presence of lower MIS-ATP, a measure of mitochondrial dysfunction caused by inhibitory substances in serum, predict the development of diabetes. If these findings are supported by ensuing additional robust reports, major attention must be given to limiting future exposure to POPs and minimizing the effect of their presence in those already exposed, as well as to producing a greater understanding of the causes of mitochondrial dysfunction.

## Data Availability

The datasets generated and/or analysed during the current study are available from the corresponding author on reasonable request.

## Abbreviations

AhR: Aryl hydrocarbon receptor
AhRL: AhR ligand bioactivity
ATP: Adenosine triphosphate
BMI: Body mass index
CSS: Charcoal-stripped human serum
ELSA-Brasil: Brazilian longitudinal study of adult health
FI: Fold Induction
FPG: Fasting plasma glucose
HbA1c: Glycated haemoglobin
HR: Hazard ratio
MIS: Mitochondria-inhibiting substances
MIS-ATP: Intracellular ATP content in serum-incubated cultured cells
OGTT: Oral glucose tolerance test
POPs: Persistent organic pollutants
TCDD: 2,3,7,8-tetrachlorodibenzo-p-dioxin
WHR: Waist/hip ratio
